# Jasmonic Acid Modulates the Morphological, Yield, and Phytochemical Responses of Roselle (*Hibiscus sabdariffa* L.) Under Drought Stress

**DOI:** 10.1002/fsn3.71054

**Published:** 2025-10-06

**Authors:** Warqaa Muhammed ShariffAl‐Sheikh, Mohamed Baqer Hussine Almosawi, Heidar Meftahizade, Seyedeh‐Somayyeh Shafiei‐Masouleh

**Affiliations:** ^1^ Faculty of Basic Science Branch, Faculty of Dentistry University of Al‐Qadisiyah Diwaniyah Iraq; ^2^ College of Education for Pure Science Al‐Muthanna University Samawah Iraq; ^3^ Department of Horticultural Science, Faculty of Agriculture & Natural Resources Ardakan University Ardakan Iran; ^4^ Department of Genetics and Breeding Ornamental Plants Research Center (OPRC), Horticultural Sciences Research Institute (HSRI), Agricultural Research, Education and Extension Organization (AREEO) Mahallat Iran

**Keywords:** abiotic stress, antioxidant activity, calyx yield, chlorophyll content, harvest index, plant hormone

## Abstract

Roselle ( *Hibiscus sabdariffa* L.) is a tropical and subtropical annual plant valued for its nutritional and therapeutic uses, including sour beverages, jams, and traditional medicines. Drought stress is a major abiotic factor limiting plant growth, yield, and quality. Jasmonic acid (JA), a key signaling molecule, plays a vital role in enhancing plant tolerance to environmental stress by supporting growth, nutrient uptake, photosynthesis, and biochemical defense. This study aimed to assess drought stress effects on roselle growth and productivity and evaluate JA's role in mitigating drought‐induced damages. A field experiment was conducted with four irrigation levels (100%, 70%, 40%, and 25% field capacity (FC)) and foliar JA applications (0, 10, 30, and 60 mg L^−1^). JA positively influenced morpho‐physiological and yield traits. The tallest plants (233.67 cm and 231.67 cm) were recorded under 70% FC with 30 and 60 mg L^−1^ JA. The highest biomass (27,060 kg/ha) was under full irrigation without JA. The greatest seed yield (1116.2 g/plant) and calyx yield (1081 kg/ha) occurred under 70% FC with 60 and 30 mg L^−1^ JA, respectively. JA improved chlorophyll content under severe drought (25% FCs) by up to 28.87%, and phenol content under well‐watered conditions by 22.73%. The highest ascorbic acid (19.04%) was observed under 25% FC + 10 mg L^−1^ JA. These results highlight the multifaceted role of JA in enhancing roselle drought tolerance and productivity, particularly under moderate water‐deficit conditions.

## Graphical abstract

The maximum fruit length in Rosella was recorded under 25% FC when treated with 10 mg L^−1^ JA. Foliar spraying of JA effectively mitigated drought‐induced reductions in plant biomass, calyx yield, and chlorophyll content. Under 25% of FC conditions, JA 10 mg L^−1^ caused a significant increase by 19.04% in the ascorbic acid content.

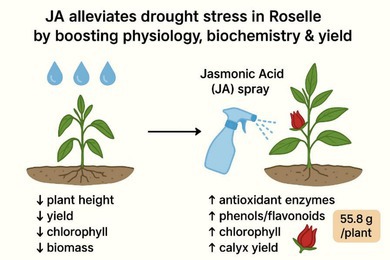

## 1 Introduction

*Hibiscus sabdarifa* L., also known as roselle, is a tropical/subtropical, annual plant with a wide variety of uses as a useful plant with quality products (Al‐Mohammad et al. 2021; Jirakiattikul et al. 2021; Mohamed et al. 2015; Mehar‐un‐Nisa Narejo et al. 2016). Its red calyx is made into sour beverages, jams, and jellies, and is understood to have medicinal properties including lowering blood pressure, fever, liver health, and cosmetic use. Roselle is included among some traditional medicines used for treating kidney stones, cholesterol issues, and infections, since it is high in vitamins, minerals, antioxidants, and bioactive compounds that are proven nutritional and therapeutic. The main anthocyanins are cyanidin‐3‐sambubioside and delphinidin‐3‐sambubioside (Mehar‐un‐Nisa Narejo et al. 2016). This plant is grown for its seeds, calyxes, stems, and leaves, and is valuable in food, medicinal, and industrial purposes (Aishah et al. 2019; Chew et al. 2024).

Drought is one of the most important abiotic stresses on the growth, yield, and quality of medicinal plants like roselle, and it is increasing due to climate change and increasing temperature. Photosynthesis, a significant parameter at the physiological level, is also severely influenced by drought. The closure of stomata decreases the availability of CO_2_, leading to overreduction of the electron transport chain of photophosphorylation, resulting in the production of reactive oxygen species that can affect the integrity of cellular structures and are wasteful to plant productivity (Mehar‐un‐Nisa Narejo et al. 2016).

Plant hormones like salicylic acid and jasmonic acid (JA) can be used in foliar applications to improve quantitative and qualitative traits to ameliorate these effects. Therefore, applying these treatments helps sustain the performance of medicinal plants in water‐limited settings (Fathi and Bahamin 2018). The progressive use of knowledge of the physiological mechanisms for drought tolerance can provide resilience to crop varieties. Recently, plant hormones such as JA have been more prominently recognized as drivers and regulators of stress responses in plants (Ahmad Lone et al. 2022). Jasmonates in regulating plant metabolism during times of stress can produce beneficial compounds depending on factors like concentration, timing of application, and plant growth stage (Aishah et al. 2019). Jasmonate levels in different plant species increased rapidly, which shows jasmonates play important roles in inducing plant defenses to biotic and abiotic challenges when experiencing drought stress, wounding, and pathogen attacks. Jasmonates induce the production of protective proteins. Moreover, exogenous JA application has been proven to increase plant drought stress tolerance (Gao et al. 2004). JA functions as an important internal signal that stimulates plant responses to abiotic stresses. Plant stresses such as drought initiate different defenses regulated through JA; for example, increases in antioxidant enzymes, an increase in specific sugars and amino acids, and reductions in water loss and stomatal behavior. On the molecular level, JA regulates specific gene expression and interacts with other plant hormones and transcription factors, providing a large network with potential for regulatory control over the metabolic adaptation of plants for growth in harsh environments (Wang et al. 2020). JA has been reported to enhance plant defenses under drought scenarios by enhancing antioxidant processes while decreasing oxidative damage (Waheed et al. 2022). The properties of JA help mitigate drought tolerance by modulating internal signaling and physiological responses, including stomatal regulation and protective gene activation.

Research on a number of species has shown that both endogenous (naturally synthesized) and exogenous applied JA can support water conservation, energy balance, and antioxidant capacity in plants (Wang et al. 2020). The growth parameters (plant height, branches number, leaves area, and chlorophyll) and yield parameters (fruits number, fresh weight calyces, fresh yield calyces, and dry yield calyces) of roselle were increased due to foliar application of Phe + JA + Bio (Al‐Mohammad et al. 2021). The foliar application of JA to the *C. roseus* plants increased the growth parameters and mitigated the salt stress; moreover, it enhanced nutrient uptake. Spraying of *C. roseus* plants with JA significantly enhanced the biosynthesis of compatible solutes and decreased the activity of pyrogallol peroxidase (PPX) and polyphenol oxidase (PPO). Foliar application of JA increased the alkaloid yield of *C. roseus* plants (Al‐Huqail and Ali 2021). Despite the growing evidence supporting JA's role in drought tolerance, studies on its specific effects in roselle remain limited. Thus, further research is needed to explore how JA influences its growth and secondary metabolism of roselle under water‐deficit conditions. Understanding this relationship can contribute to the development of agronomic practices aimed at enhancing drought resilience and improving the qualitative and quantitative traits of roselle. This study aimed to: (i) assess the impact of drought stress on growth and productivity parameters of roselle, and (ii) evaluate the effectiveness of JA in mitigating drought‐induced damage. To achieve these goals, a field experiment was conducted using roselle plants subjected to four irrigation regimes and three foliar sprays of JA.

## 2 Materials and Methods

**FIGURE 1:**
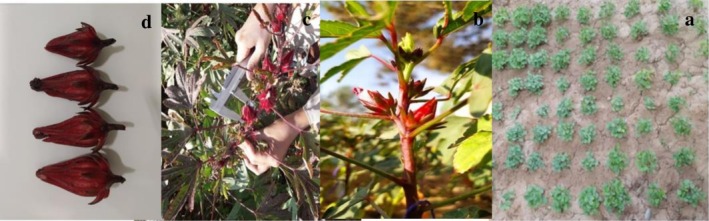
Rosella plantation and measurements of morpho‐physiological characteristics. (a) Sowing Rosella seeds, (b) Flowering phase and application of jasmonic acid under drought stress, (c) Measuring traits in different stages, (d) Production of Rosella boll under drought stress by jasmonic acid.

An experiment was designed and conducted in a factorial design within a randomized complete block design (RCBD) with three replications and two factors under study: irrigation at 4 levels ((control = 100% field capacities (FCs)), 70% FC, 40% FC, and 25% FC) and JA was foliar‐applied at 0, 10, 30, and 60 mg/L after 20 days from seed sowing and spread six times at 15‐day intervals in the research greenhouse of Al‐Muthanna University, located in Al‐Muthanna Governorate in southern Iraq, Iraq, in 2023–2024 (Figure 1). The total duration of the experimental period was 4 months. The control treatment was 100% FC without JA.

### 2.1 Soil Characterization

The soil texture was sandy loam (0.83 ds m^−1^, pH 7.1) with organic matter content of 7.8 g kg^−1^ soil, FC of 28.7%, total nitrogen (N_kjeldhal_) of 0.02%, available phosphorus (P) of 16 mg^−1^ kg^−1^ soil, and available potassium (K) of 211 mg^−1^ kg^−1^.

### 2.2 Seed Cultivation and Fertilizer Application

Three seeds were sown by hand per hole. About 25 kg ha^−1^ P_2_O_5_ was added to the seedbed before planting, and 30 kg ha^−1^ nitrogen (ammonium nitrate) was applied at 40 DAP to meet the nutrient requirements of plants. Weed control was achieved manually at 25 and 50 DAP. Water stress was applied during the experiment (4 months) using four gradient levels.

The soil moisture content in each plot was daily measured by means of a time‐domain reflectometry sensor from a 20 cm soil layer. The depth of water (*d*_*iw*_) required to bring the moisture content of the soil back to the FC was assessed according to the following equation (Equation 1) (Cuenca 1989):

(1) $$d_{\mathrm{iw}}=\left(\mathrm{PFC}-\mathrm{Pi}\right)\cdot D\cdot B_{d}$$

PFC and Pi are water content at FC and at the time before irrigation (in terms of weight percentage), respectively, *B*_*d*_ is bulk specific gravity in g cm^−3^, and *D* is effective root depth in cm. The total volume of irrigation water in the scale of plot area was determined after calculation of *d*_*iw*_. Irrigation at each stage was performed when the soil's moisture content was not more than FC's.

### 2.3 Assessment of Morphological Parameters and Yield

The PH: plant height (cm), BRP: number of branches per plant, FL: fruit length (cm), BOP: number of bolls per plant, SFW: weight of sepal fresh per plant, CY: calyx yield/ha (kg), SC: number of seeds per capsule, SP: number of seeds per plant, 1000SW: thousand seed weight (gr), BY: biomass yield per hectare (kg), and HI: harvest index (%) were determined at the physiological maturity stage, when the calyx is red and the seeds are brown (Figure 1). The sepal sampling was performed at the maturity stage (browning seeds). The dried sample of calyx was prepared at room temperature for the chemical analyses.

### 2.4 Determination of Phytochemical Parameters

#### 2.4.1 Total Phenolic Content

The protocol developed by Papoti and Tsimidou (2009) was employed to assess the total phenolic content. Roselle dried sepal (500 mg) was extracted with 20 mL of methanol (with a distilled water ratio of 8:2 v/v) using 10 min of room temperature ultrasonic. The extraction process continued for another 20 h in darkness and at room temperature. The methanol extracts were centrifuged at 4000 rpm for 15 min. Folin Ciocalteu (Merck, Germany) was used as a reactive agent. An extract aliquot (100 μL) was applied to and blended with 2 mL Folin Ciocalteu reagent (1:10 diluted with distilled water) and 2.5 mL Na_2_CO_3_ aqueous 7.5 (w/v). The total phenolic content of the resulting solutions was calorimetrically assessed at room temperature at a wavelength of 765 nm (UV 2600; Shimadzu Corporation, Kyoto, Japan) after 80 min of incubation, in which gallic acid was added as normal. Total phenolic content was measured as equivalent (g/DW) to gallic acid.

#### 2.4.2 Total Flavonoids

To evaluate the flavonoid amount, 1.5 mL of 95% ethanol, 0.1 mL of 10% aluminum chloride (AlCl_3_, 6H_2_O), 0.1 mL of sodium acetate (NaC_2_H_3_O_2_·3H_2_O) (1 mol/L), and 2.80 mL of distilled water were mixed with each 500 μL of the sample. Using a spectrophotometer, the optical intensity was measured by absorption at a wavelength of 415 nm. In a blank analysis, the same technique was used (Lin and Tang 2007). The findings were represented in milligrams of equivalent quercetine per gram of extract. The tests were done in triplicate.

#### 2.4.3 Free Radical Scavenging Activity (DPPH)

**FIGURE 2:**
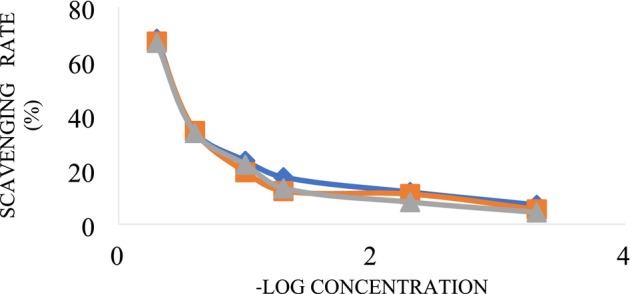
The standard curve of antioxidant activity.

The total antioxidant capacity of fruit tissues was evaluated using the DPPH assay, as described by Gil et al. (2000). A 1 g sample of dry sepal was blended with 10 mL of 80% methanol and homogenized thoroughly. The mixture was incubated at ambient temperature for 30 min, followed by centrifugation at 4°C for 10 min to obtain the supernatant. For the assay, 100 μL of the leaf extract was mixed with 900 μL of a 500 μM DPPH solution prepared in ethanol, vigorously agitated, and incubated in darkness for 30 min. The extract was replaced with methanol as a control. This value was read at 517 nm with the use of a spectrophotometer. The standard curve of antioxidant activity is presented in Figure 2. The antioxidant activity was calculated in the assay protocol using the following Equation (2).

(2) $$\text{Antioxidant}\;\text{activity}\;\left(\%\right)=\left[1-\frac{\mathrm{AED}-\mathrm{AEM}}{\mathrm{ACD}}\right]\times 100$$

AED, absorbance of extract‐DPPH mixture; AEM, absorbance of extract‐methanol mixture; and ACD, absorbance of ethanol‐DPPH (control).

#### 2.4.4 Ascorbic Acid

0.1 g of the samples was extracted with 10 mL of 3% metaphosphoric acid solution. One mL of the resulting extract was added to 9 mL of 2,6‐dichloroindophenol and then vortexed. The samples were placed in the dark, and after 20 min, the absorbance of the samples was read with a spectrophotometer at a wavelength of 515 nm (Khazaei et al. 2023). L‐ascorbic acid was used to prepare the standard sample.

#### 2.4.5 SPAD

Chlorophyll concentration index (SPAD) was determined at 10:00 h with the SPAD‐502 on non‐sampled leaves. Measurements were performed on the second fully expanded leaf, randomly selected, with three replications per treatment. The SPAD values were measured with a SPAD‐502 chlorophyll meter to take accurate and repeatable measurements.

### 2.5 Statistical Analysis

The collected data were statistically analyzed using IBM SPSS statistics software (ver. 26), Minitab software (ver. 16), and R‐studio software (ver. i386, 3.2.2). The Multiple Duncan test at the 0.05 level was used to compare means. The graphs were designed using Excel software (2010), Minitab software (ver. 16), and R‐studio software (ver. i386, 3.2.2).

## 3 Results

The ANOVA results indicated that the growth, yield, and quality of the roselle were significantly influenced by the main effects of the elicitors and irrigation regimes, as well as their two‐way interactions (Table 1).

**TABLE 1:** The results of ANOVA of morpho‐physiological, biochemical, and yield attributes of roselle plants in response to drought stress and JA application.

Source	df	PH	BRP	FL	BOP	SFW	CY
Drought (FC)	3	824.9*	12.08^ns^	0.54^ns^	3097.3**	745.81**	92,490.6*
JA	3	3019.99**	18.63*	0.19^ns^	594.72**	83.25^ns^	10,500.5^ns^
FC × JA	9	478.09^ns^	6.93^ns^	4.31*	323.76**	115.72^ns^	29,544.1^ns^
Error	24	321.11	5.46	1.97	79.88	154	21,070.1
CV%		8.80	13.06	19.01	12.74	31.96	16.39

Source	df	SC	SP	1000SW	BY	HI	
FC	3	22.96**	45,946.0**	46.90*	1,267,008**	2.20*	
JA	3	6.427	20,181.5^ns^	1.80^ns^	215,514.7^ns^	0.09^ns^	
FC × JA	9	4.52 ns	49,126.1^ns^	27.90^ns^	508,348.0^ns^	0.31^ns^	
Error	24	5.09	35,308.7	13.94	2,651,367	0.53	
CV%		21.33	24.11	12.75	6.51	19.40	

Source	df	Flavonoid	Phenol	Antioxidant	Ascorbic acid	SPAD	
FC	3	60.78**	0.87**	14.99**	436.46 ns	256.79**	
JA	3	83.62*	0.48^ns^	10.58**	416.80 ns	46.91**	
FC × JA	9	71.17*	0.69^ns^	12.29**	832.70**	36.01**	
Error	24	23	0.49	1.49	209.84	7.642	
CV%		4.72	1.56	3.62	10.29	4.53	

*Note:* ** and * show the significant levels at *p* ≤ 0.01, and *p* ≤ 0.05, respectively. Color bar displays correlation coefficient values (−1 to +1), the reds show positive relationships, and the blues show negative relationships.

Abbreviations: 1000SW, thousand seed weight; BOP, boll/plant; BRP, branch/plant; BY, biomass yield/ha; CY, calyx yield/ha; FC, field capacity; FL, fruit length; HI, harvest index; JA, jasmonic acid; ns, not significant; PH, plant height; SC, seeds/capsule; SFW, sepal fresh weight/plant; SP, seed/plant; SPAD, chlorophyll.

### 3.1 The Morpho‐Physiological and Yield

**FIGURE 3:**
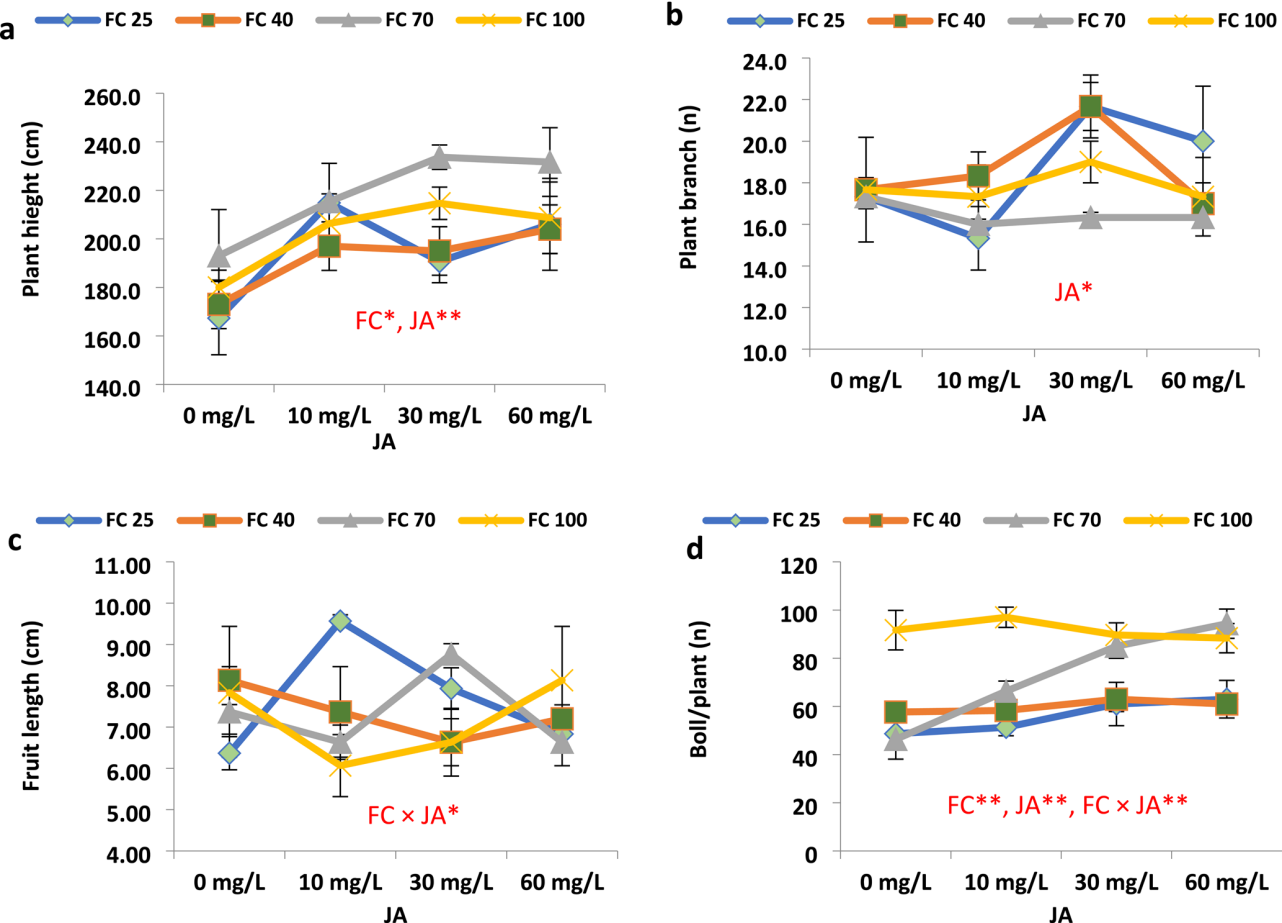
Jasmonic acid modulation of some morpho‐physiological variables of roselle plants under different field capacities (FC). (Duncan test, *n* = 3, *α* = 0.05).

**FIGURE 4:**
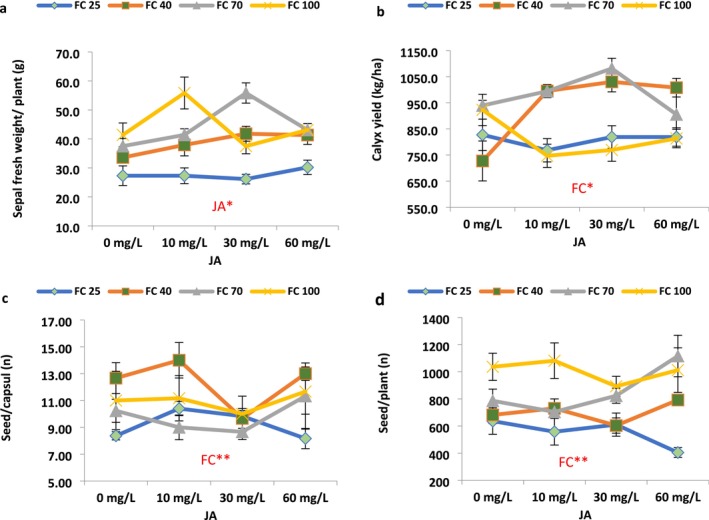
Jasmonic acid modulation of some seed yield and calyx yield components in the roselle plants under different field capacities (FC). (Duncan test, *n* = 3, *α* = 0.05).

**FIGURE 5:**
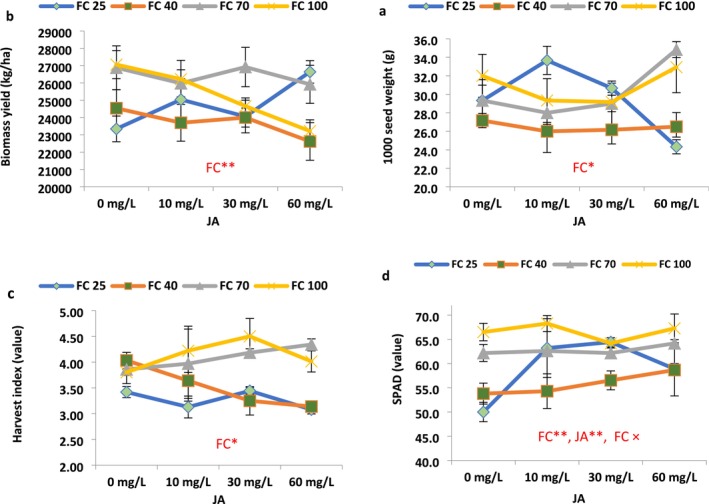
Jasmonic acid modulation of plant yield, seed weight, harvest index, and SPAD value in the roselle plants under different field capacities (FC). (Duncan test, *n* = 3, *α* = 0.05).

Drought stress significantly affected plant height, boll per plant, sepal fresh weight, calyx yield, seeds per capsule, seed yield, 1000 seed weight, biomass yield, and harvest index (HI). JA levels had significantly influenced plant height, branch per plant, and boll per plant. The interaction effect of irrigation and JA levels was significant for fruit length, boll per plant, flavonoid, antioxidant, ascorbic acid, and chlorophyll index (SPAD) (Figures 3, 4, 5).

Overall, the values of plant height, boll per plant, seed per plant, biomass yield, HI, and chlorophyll content were significantly diminished in response to irrigations of 40% and 25% FC (high drought stress) compared to FC irrigation (Figures 3, 4, 5).

Low drought stress (70% FC irrigation) significantly increased roselle plant height compared to fully irrigated conditions (100% FC), while a significant decrease was observed at 25% FC (Figure 3a). Notably, the highest plant height (233.67 and 231.67 cm) was recorded under 70% FC irrigation when treated with 30 and 60 mg L^−1^ JA foliar spray, which effectively alleviated the stress effects and prevented a reduction in plant height. Under a 40% irrigation regime, the highest plant height reached 204.0 cm in response to 60 mg/L JA, while at 25% FC irrigations, with 10 and 60 mg L^−1^ JA, the plant height reached 214.83 and 206.0 cm, respectively, indicating a substantial 28.38% and 23.10% increase compared to the control (167.3 cm) (Figure 3a).

Drought stress led to a nonsignificant gain in the number of branches per plant. However, the maximum number of branches per plant (21.7) was achieved under 40% and 25% irrigations with 30 mg L^−1^ JA, demonstrating significant 22.64% and 25.04% increases compared to the control (17.7) (Figure 3b).

Regarding fruit length, the maximum fruit length (9.57 cm) was recorded under 25% FC when treated with 10 mg L^−1^ JA, which effectively mitigated the stress effects and prevented a severe decline in fruit length. Under these stressed conditions (25% FC), JA 10 and 30 mg L^−1^, respectively, caused significant increases of 50.26% and 24.60% in fruit length compared to untreated plants with JA (Figure 3c).

Drought stress exhibited a linear drop in the number of bolls per plant (Figure 3d). Correspondingly, the maximum number of bolls per plant (97) was obtained under well‐watered irrigation (100% FC) with 10 mg L^−1^ JA, showing a moderate 5.81% increase compared to the control (91.7) (Figure 3d). Under 25% of FC conditions, JA 30 and 60 mg L^−1^, respectively, caused notable improvements of 25.33% and 29.44% in the bolls per plant compared to untreated plants with JA (Figure 3d). In addition, in the 70% irrigation condition, bolls per plant were significantly augmented by 43.17%, 83.47%, and 103.60%, respectively, with the application of 10, 30, and 60 mg L^−1^ JA compared to non‐JA‐treated plants (Figure 3d).

Sepal fresh weight per plant only exhibited a meaningful reduction under irrigation of 25% FC, compared to its value in the well‐watered‐irrigated plants (Figure 4a). The fresh weight of calyx (sepal) per plant was significantly decreased at high drought stress conditions (25% FC). Foliar application of JA had varied impacts on this parameter, increasing it under certain conditions and decreasing it in others. The maximum fresh weight of calyx per plant (55.8 kg) was observed under moderate drought stress conditions (70% FC) with 30 mg L^−1^ JA application, indicating a significant 48.87% increase compared to the plants untreated with JA at 70% FC (Figure 4a). In the 40% irrigation of FC situation, JA 10 mg L^−1^ caused an improvement of 12.88% in the sepal fresh weight. Also, JA 30 mg L^−1^ caused a significant upsurge of 24.35% in the sepal fresh weight (Figure 4a).

Calyx yield per hectare demonstrated an increase with intensifying drought stress at 40% and 70% of FC irrigation, while a decrease was noted at 25% irrigation. The maximum calyx yield per hectare (1081 kg/h) was recorded under low drought stress conditions (70% FC) with 30 mg L^−1^ JA, representing a substantial 15.10% increase compared to the non‐JA‐treated plants (939.58 kg/h) (Figure 4b). Application of JA 10 mg L^−1^ caused a meaningful escalation of 36.80% in calyx yield in response to the 40% irrigation of FC (Figure 2b). Furthermore, the value of calyx yield was significantly increased by 38.58% in plants treated with 60 mg L^−1^ JA in response to 40% FC irrigation (Figure 4b).

The number of seeds per capsule showed an increase with increasing drought stress until 40% FC irrigation, while it reduced under 25% FC compared to control (Figure 4c). The maximum number of seeds per capsule (14.0) was recorded under drought stress conditions (40% FC) with 10 mg L^−1^ JA, demonstrating a meaningful 10.52% increase compared to the control (11.0) (Figure 4c). Under 25% of FC conditions, seeds per capsule significantly increased by 24.31% and 17.52% in response to JA 10 and 30 mg L^−1^, respectively (Figure 4c).

Furthermore, the number of seeds per plant also increased with increasing drought stress (Figure 4d). The highest seed yield per plant (1116.2 g) was achieved under drought stress conditions (70% FC) with 60 mg L^−1^ JA, indicating a substantial 42.03% significant increase compared to the control (636.3 g) (Figure 4d). The value of seeds per plant was significantly increased by 16.15% in plants treated with 60 mg L^−1^ JA in response to 40% FC irrigation (Figure 4d).

Regarding biomass yield, an initial increase followed by a decreasing trend was observed with increasing drought stress (Figure 5a). Interestingly, foliar application of JA exhibited varied effects on biomass yield, increasing it under certain conditions while decreasing it in others. The maximum biomass yield (27,060.00 kg/h) was obtained under FC irrigation without JA application. JA 60 mg/L caused notable improvements by 14.11% in the plant biomass yield in response to 25% irrigation (Figure 5a); however, in the FC100%, JA caused nonsignificantly reduced biomass yield.

The 1000‐seed weight initially displayed a constant trend with increasing drought stress (70% FC); however, it exhibited a downward trend with further application of stress (40% and 25% FC irrigations). The use of JA showed a capacity to reduce stress effects in some instances while having a negative impact under specific stress conditions. Under 25% of FC conditions, JA 10 mgL^−1^ caused a significant increase of 14.78% in the 1000 seed weight (Figure 5b). The highest 1000‐seed weight (34.8 g) was observed under low stress conditions (70% FC) and the application of 60 mg L^−1^ JA, showing an 18.75% rise compared to non‐JA‐treated plants (29.33 g) (Figure 5b). The HI also decreased with increasing drought stress. Foliar application of JA had both nonsignificant positive and negative effects on the HI. The highest HI (4.5) was observed in non‐stress conditions (the control plants treated with 30 mg L^−1^ JA), indicating a nonsignificant increase of 18.36% compared to the non‐JA‐treated plants (3.4) (Figure 5c). In the 40% FC irrigation, JA 30 mg L^−1^ caused a nonsignificant reduction in the HI compared to non‐JA‐treated plants (Figure 5c).

Overall, the application of JA demonstrated additive effects on the chlorophyll value in roselle plants under different levels of irrigation. Under well‐watered conditions and low and moderate drought stress (70% and 40% irrigations), the application of JA nonsignificantly increased chlorophyll content; however, in the high stress condition (25% FC), chlorophyll content was improved remarkably with the application of all JA levels compared to non‐JA‐treated plants (Figure 5d), as it was increased by 26.40%, 28.87%, and 17.80% with the application of 10, 30, and 60 mg L^−1^ JA, respectively (Figure 5d).

### 3.2 Physio‐Biochemical Characteristics

**FIGURE 6:**
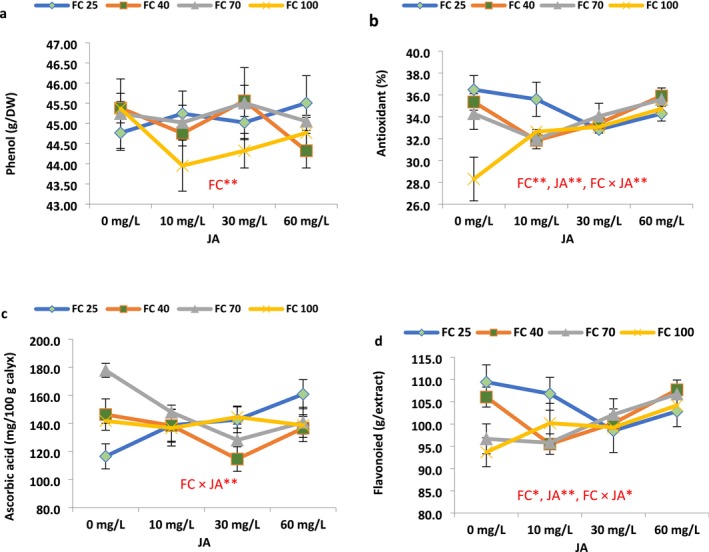
Jasmonic acid modulation of some biochemical and photosynthetic characteristics in the roselle plants under different field capacities (FC). (Duncan test, *n* = 3, *α* = 0.05).

Drought stress significantly affected flavonoid, phenol, ascorbic acid, and antioxidant contents, in addition to flavonoid content. JA levels had a significant influence on flavonoid and antioxidant capacity. The interaction effect of irrigation and JA levels was significant for flavonoid, antioxidant, ascorbic acid, and flavonoid content (Figure 6).

Drought stress caused significant increases in the phenol content of roselle plants; however, the effect of JA levels was not significant. The application of JA at different levels showed varied nonsignificant effects on the phenol content (g DW) in roselle plants under different irrigation regimes (Figure 6a). The antioxidant activity was significantly increased in response to all drought treatments (Figure 6b). The effect of JA on the antioxidant percentage in roselle plants under drought stress proved to be complex, depending on both the stress level and the applied JA concentration (Figure 6b). Under well‐watered conditions (100% FC irrigation), 10, 30, and 60 mg L^−1^ JA, respectively, led to significant increases of 15.31%, 16.88%, and 22.73% compared to control; however, under severe drought stress (25% and 40% irrigation), JA resulted in a non‐notable decrease in antioxidant percentage.

The effect of JA on the ascorbic acid content under drought stress displayed variable patterns, strongly influenced by both the stress level and the JA concentration. Under well‐watered conditions (100% FC irrigation), JA did not significantly affect ascorbic acid content; however, it led to a significant reduction in ascorbic acid content in response to moderate drought stress (70% irrigation) and a noteworthy increase in response to severe drought stress (25% FC). Under 25% of FC conditions, JA 10 mgL^−1^ caused a significant increase of 19.04% in the ascorbic acid content (Figure 6c). At the 40% irrigation level, JA 30 mg L^−1^ caused a significant reduction in the ascorbic acid compared to non‐JA‐treated plants (Figure 6c).

Different levels of JA and irrigation exhibited varying effects on the flavonoid content in roselle plants. Increasing drought stress resulted in raising flavonoid content in a linear trend with a low slope. Under well‐watered conditions (100% FC), the application of JA resulted in a nonsignificant rise in flavonoid content. In the low and moderate drought stress, the application of lower JA concentrations (10 and 30 mg L^−1^) appeared to induce an increase in flavonoid content. However, under the most severe drought stress (25% FC), a consistent trend was evident, and different JA levels showed negative effects (Figure 6d). Overall, the response of flavonoid content to JA application seems to be intricately linked to the severity of drought stress, without displaying a linear or uniform pattern (Figure 6d).

### 3.3 Principal Component Analysis

**FIGURE 7:**
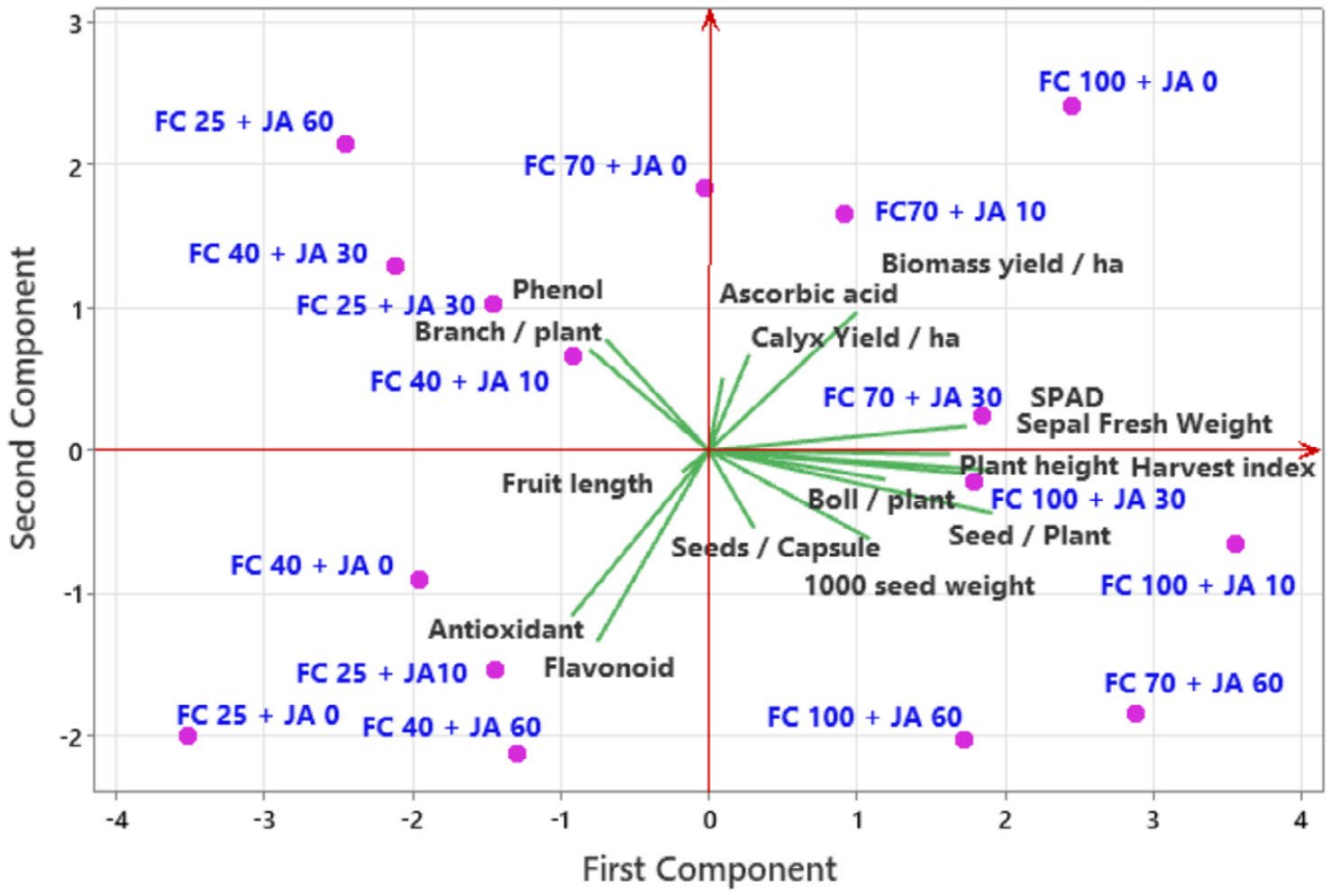
PCA diagram of morpho‐physiological, yield, and physio‐biochemical traits in the roselle plants under different field capacities (FC) and jasmonic acid levels (JA).

Principal component analysis (Figure 7) revealed a total of 73% variance between variables (first component 0.57 and second component 0.16 with an eigenvalue of 4.37 and 2.51, respectively). PCA analysis revealed different trends between variables and treatments, SPAD value highly correlated with 70% of FC irrigation × 30 mg L^−1^ JA, and showed a unique trend (quarter 1). Biomass yield was highly correlated with FC 70% × JA 10 mg L^−1^ (quarter 1), while calyx yield and ascorbic acid were less correlated to this treatment. Overall, the morpho‐physiological and yield attributes were highly associated with non‐drought stress condition and application of 10, 30, and 60 mg L^−1^ JA, and 70% FC irrigation with 60 mg L^−1^ JA (quarter 4). The values of antioxidant and flavonoid (quarter 3) were significantly related to high drought stress (FC 25%); and FC 25% + application of 10 mg L^−1^ JA, as well as FC 40% + application of 60 mg L^−1^ JA. Phenol content and branch number were significantly correlated to FC 25% + 30 mg L^−1^ JA; and FC 40% + 10 and 30 mg L^−1^ JA (quarter 2).

### 3.4 Pearson's Correlation

**FIGURE 8:**
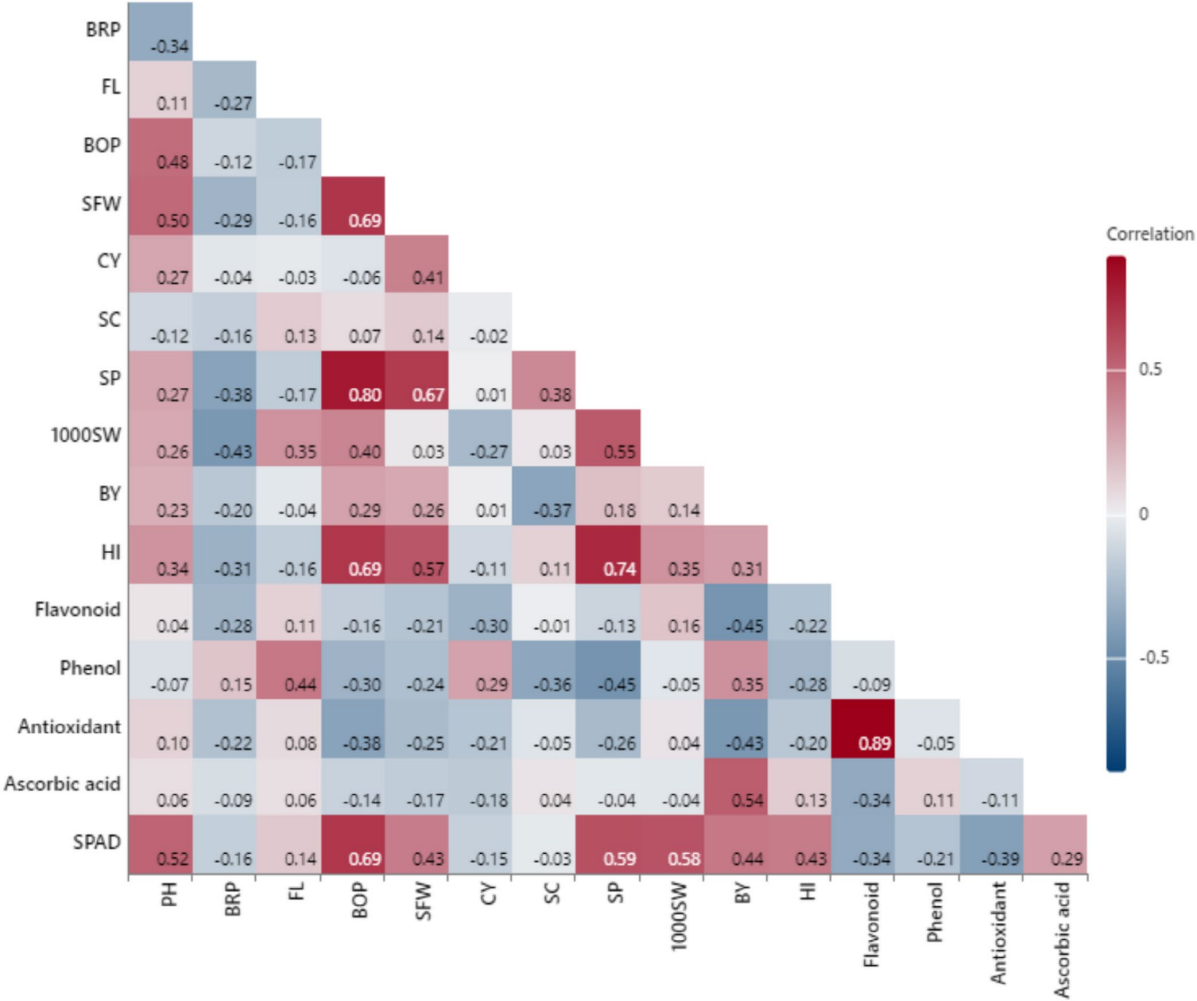
Correlation matrix illustrating the relationships between morpho‐physiological traits, yield components, and physio‐biochemical attributes of *Hibiscus sabdariffa* L. under drought stress and jasmonic acid application. 1000SW, thousand seed weight; BOP, boll per plant; BRP, branch per plant; BY, biomass yield per ha; CY, calyx yield per ha; FL, fruit length; HI, harvest index; PH, plant height; SC, seeds per capsule; SFW, sepal fresh weight per plant; SP, seed per plant; SPAD, chlorophyll. Color bar displays correlation coefficient values (−1 to +1), the reds show positive relationships, and the blues show negative relationships.

The correlation analysis (Figure 8) revealed several significant relationships between yield components, morpho‐physiological, and biochemical attributes, including a positive correlation between sepal fresh weight (SFW) and boll/plant (BOP) (*r* = 0.69*); a strong positive correlation between SP and boll/plant (BOP) (*r* = 0.80**), and SFW (*r* = 0.67*). Similarly, a positive correlation between HI and boll/plant (BOP) (*r* = 0.69*); and SP (*r* = 0.74**). Furthermore, SPAD value showed a positive correlation with boll/plant (BOP) (*r* = 0.69*), indicating that plants with more chlorophyll content tend to produce higher boll yields. The strong positive correlation between HI and seed yield per plant (seed/plant) highlights that plants efficiently partition dry matter into seeds exhibit higher yields. Regarding biochemical attributes, positive correlations were found between flavonoid content and antioxidant percentage (*r* = 0.89**). These associations suggest that an upsurge in flavonoid content is generally accompanied by an increase in antioxidant activity, potentially supporting the role of flavonoid compounds in the plant's defense mechanisms against drought stress.

### 3.5 Regression

**FIGURE 9:**
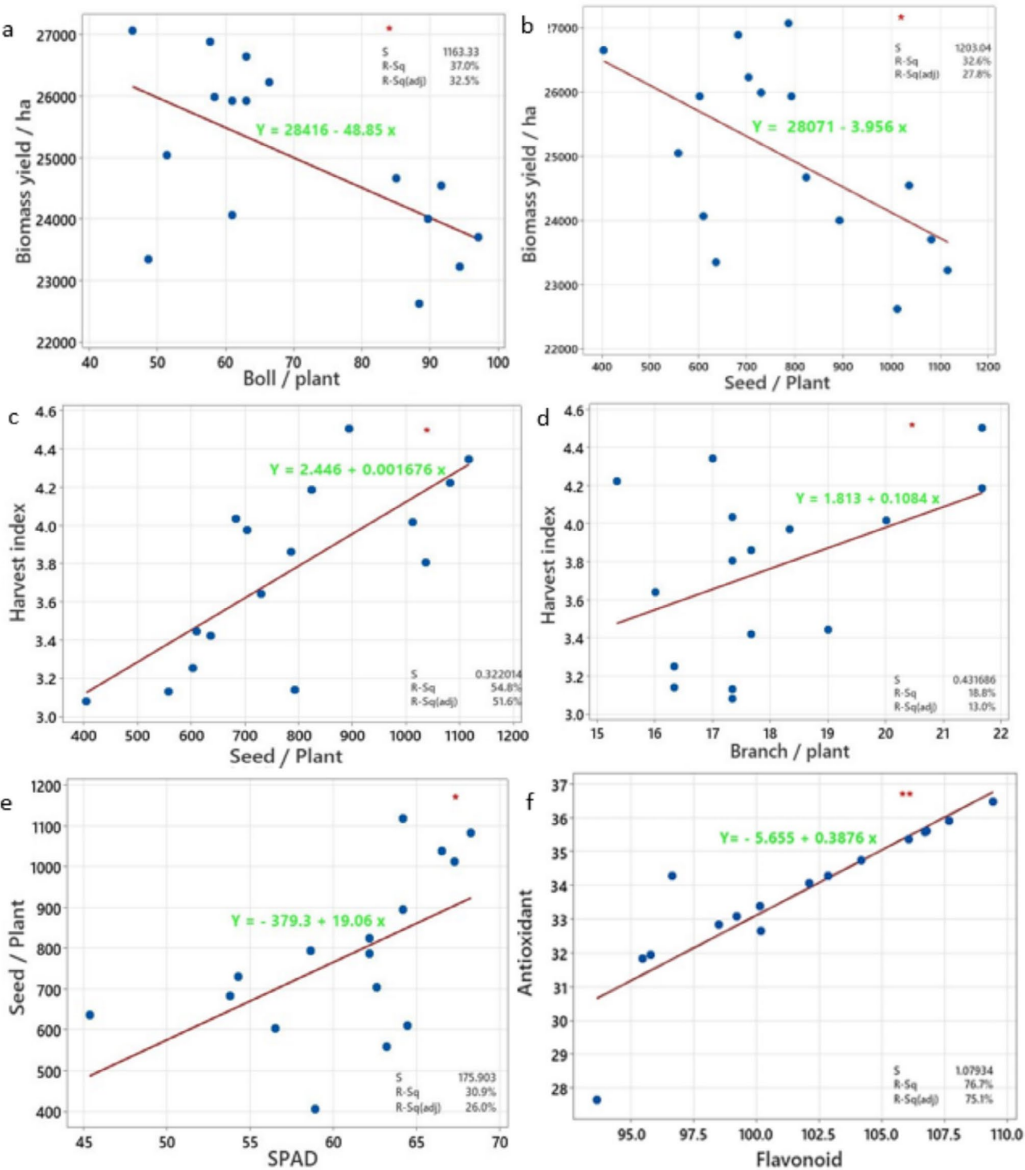
(a) Linear regression diagram of biomass and boll/plant, (b) biomass and seed/plant, (c) harvest index and seed/plant, (d) harvest index and branch/plant, (e) seed production and SPAD value, and (f) antioxidant activity and flavonoid content in the roselle plants under different irrigation × Jasmonic acid levels (JA).

The results of the linear regression analysis (Table 2) revealed that the traits boll per plant and seed per plant exhibited significant variance with plant biomass (dependent variable) at the level of 5%. The variables branch per plant and seed per plant showed significant variances with HI (dependent variable). Also, SPAD value (predictor) exhibited a significant variance with seed per plant as the dependent variable. Furthermore, the variance between flavonoid content (predictor) and antioxidant percent was significant at the level of 1%. Consequently, corresponding regression plots were generated (Figure 9).

**TABLE 2:** Results of ANOVA for linear regression between variables.

Source	df	Plant biomass^D^ − boll per plant^P^		Plant biomass^D^ − seed per plant^P^		Harvest index^D^ − branch per plant^P^	
		MS	*p*	MS	*p*	MS	*p*
Regression	1	11,128,820	0.012	9,813,238	0.021	0.604548	0.013
Error	14	1,353,340		1,447,310		0.186353	

Source	df	Harvest index^D^ − seed per plant^P^		Seed per plant^D^ − SPAD^P^		Antioxidant^D^ − flavonoid^P^	
		MS	*p*	MS	*p*	MS	*p*
Regression	1	1.76178	0.001	193,835	0.025	53.76	0.0001
Error	14	0.10369		30,942		1.17	

*Note:* ^D^Dependent variable, ^p^Predictor.

The linear regression analysis between plant biomass (dependent variable) and bolls per plant (independent variable) (Figure 9a) indicated a significantly negative linear relationship. The adjusted coefficient of determination (*R* ^2^ adj) for this regression was 32.5%, and the fitted linear equation was: *Y* = 28,416 – 48.85 *X*. The linear regression analysis between plant biomass (dependent variable) and seed per plant (independent variable) (Figure 9b) showed a significant negative linear relationship, indicating that plant biomass decreased with increasing number of seeds per plant. The adjusted coefficient of determination (*R* ^2^ adj) for this regression was 27.8%, and the fitted linear equation was: *Y* = 28,071 – 3.96 *X*. The linear regression for HI (dependent variable) and seed per plant (independent variable) presented a significantly positive linear relation (Figure 9c) as the adjusted coefficient of determination (*R* ^2^ adj) was 51.6%, and the fitted linear equation was: *Y* = 2.45 + 0.002 *X*.

As shown in Figure 9d, linear regression for HI (dependent variable) and branches per plant (independent variable) showed a significantly positive linear association. The adjusted coefficient of determination (*R* ^2^ adj) for this linear regression was 13.0%. The fitted linear equation was: *Y* = 1.813 + 0.11 *X*.

As presented in Figure 9e, the linear regression between seed per plant (dependent variable) and SPAD value (independent variable) displayed a significantly positive linear association. The fitted linear equation was: *Y* = −379.3 + 19.06 *X* and the adjusted coefficient of determination (*R* ^2^ adj) for this linear regression was 26.0%.

Finally, the linear regression analysis between antioxidant percent (dependent variable) and flavonoid content (independent variable) (Figure 9f) revealed a significantly positive linear relationship. The adjusted coefficient of determination (*R* ^2^ adj) for this regression was 75.1%, and the fitted linear equation was: *Y* = −5.66 + 0.39 *X*.

## 4 Discussion

Drought stress in roselle resulted in varying responses that included mild stress (70% FC), which proved positive for several growth and yield variables, including plant height, biomass, and number of seeds; to severe drought stress (25% FC), with which reductions occurred in most variables. However, foliar applications of JA were able to ameliorate the impacts of drought stress using concentrations of 10–60 mg L^−1^ JA as a beneficial treatment. JA dramatically increased plant height, number of branches and bolls, length of fruit, seeds and calyx yield, and HI in plants under severe drought (FC25%). These results indicate the positive role that JA plays in increasing stress tolerance.

Given water scarcity in the cultivation regions of roselle, applying 75% of the crop's water needs helps conserve resources (Albalsmeh and Piri 2024). Reduced irrigation (2 and 4 h weekly) led to notable decreases in seed and biological yields, up to 49% and 48% in roselle, respectively (Mehrnia et al. 2024). Fallahi et al. (2016) observed that drought stress significantly reduced plant growth and yield traits. A field experiment (Zand‐Silakhoor et al. 2023) to assess the effects of irrigation intervals on roselle yield and water use efficiency showed that irrigation significantly influenced most growth traits. A greenhouse study evaluated the impact of two irrigation levels on the growth and phenolic content of *H. sabdariffa* var. UKMR‐2. Plants irrigated with 1.72 L/day showed slightly higher growth performance, total phenolic content, anthocyanin levels, and antioxidant activity compared to the lower irrigation treatment. Despite this, 1.72 L/day irrigation demonstrated better overall quality and yield, making it the more suitable irrigation level for optimizing roselle phenolic production without compromising plant health (Ali et al. 2019). In a greenhouse study, roselle plants subjected to drought stress (25% soil saturation) showed notable reductions in height, flower number, and photosynthetic rate compared to fully watered controls. Shala and Mahmoud (2018) showed that severe water stress (80% depletion) significantly reduced vegetative traits, fruit number, fresh and dry calyx weights, seed yield, and overall productivity.

JA and methyl jasmonate are natural hormones affecting developmental processes and responses to stress in plants. They can affect physiological processes such as flowering, photosynthesis, and seed germination. Understanding how JA influences development is important, especially for increasing yield and improving quality during environmental stressors like drought (Mehrnia et al. 2024). Recently, JA has emerged as an excellent growth hormone to improve drought tolerance owing to its involvement in different plant physiological and biochemical processes (Baek et al. 2020). JA improves membrane stability, plant water relations, nutrient uptake, osmolyte accumulation, and antioxidant activities that can counter the toxic effects of drought.

JA can also have roles in developmental processes, such as root elongation and aging. JA signals genes to produce proteins, such as proteinase inhibitors and other molecules that enhance plant resistance to stress conditions (Hassanein et al. 2009). Alam et al. (2014) have explored the basic role of JA in inducing drought tolerance in three Brassica species and stated that JA can delay the negative effects of drought on the plants' growth by modulating their antioxidant responses and improving their water status (Alam et al. 2014).

It also contributes to signaling pathways, i.e., gene networks, stress‐responsive proteins, signaling intermediates, and enzymes that protect the plants from the toxic effects of drought (Khan et al. 2024). In the JA signaling pathway, the involvement of JAZ in drought tolerance has been reported. Overexpression of OsJAZ9 reduced leaf width and stomatal density, thereby lowering the leaf transpiration rate and improving rice tolerance to water‐deficit stress (Singh et al. 2021). Further, a large set of JA‐responsive genes was induced under drought stress in barley (Svoboda et al. 2016). It was also observed that the foliar pretreatment with MeJA positively impacted drought tolerance in maize (Tayyab et al. 2020). Recently, direct evidence of JAs' role in drought emerged in Arabidopsis overexpressing a JA signaling repressor gene, AtJAZ7. Furthermore, overexpression of the TIFY transcription factor ZmJAZ13 displayed drought tolerance by modulating redox balance, defense metabolites, and photosynthesis (Zhang et al. 2025). Exogenous application of acetic acid also conferred drought tolerance in plants by promoting JA signaling (Kim et al. 2017). Furthermore, JA is organized with many other hormones, while it acts primarily to enhance cellular defense and ensure plant health during stress; it also provides an opportunity for improving crop resilience under adverse conditions (Raza et al. 2021).

JA application had dose‐dependent effects on antioxidant and pigment traits in roselle. The highest chlorophyll levels were observed with 10 mg L^−1^ JA in the control plants (100% FC irrigation). Also, in the high stress condition (FC25%), chlorophyll content was improved remarkably with the application of all JA levels compared to non‐JA‐treated plants, suggesting its role in preserving photosynthetic capacity under stress. In addition, under 25% of FC irrigation, JA 10 mg L^−1^ caused a significant increase in the ascorbic acid content; however, in the 40% irrigation level, JA 30 mg L^−1^ caused a significant reduction in the ascorbic acid compared to non‐JA‐treated plants. Furthermore, in the low and moderate drought stress, the application of lower JA concentrations (10 and 30 mg L^−1^) appeared to induce an increase in flavonoid content. However, under the most severe drought stress (FC25), different JA levels showed negative effects. These results highlight the importance of a specific JA dose for improving drought resistance in roselle.

A recent study on *Grewia asiatica* JA‐treated plants showed a 15.5% increase in growth and significantly higher CO_2_ assimilation and stomatal conductance compared to untreated plants. Additionally, total phenolics and antioxidant levels increased by 34% and 63% (Waheed et al. 2022). Sheyhakinia et al. (2020) showed that JA was important for helping Roselle cope with salinity by increasing plant growth, enhancing photosynthetic pigments, and antioxidant activity. In addition, JA increased leaf traits and total phenols, flavonoids, and proline accumulation under salinity, where all contributed to improved stress tolerance.

In the *Brassica rapa* , KS101 and KBS3 genotypes, JA improved key physiological traits, including photosynthetic rate, pigment content, stomatal conductance, osmolyte accumulation, reduced membrane damage, and antioxidant activity (Ahmad Lone et al. 2022). In wheat plants, 100 μM JA and 10 mM SA have improved drought tolerance and promoted growth (Ilyas et al. 2017).

The correlation and regression analysis highlights key yield‐related traits such as sepal fresh weight, boll per plant, seed yield per plant, and SFW, seed yield, HI, and SPAD value as strong predictors of plant yield, emphasizing their role in enhancing overall productivity. Positive correlations among biochemical traits suggest a coordinated biochemical defense response to drought stress. Additionally, the inverse relationship between plant biomass, boll per plant, and seed weight reflects a resource allocation strategy that influences yield composition under limited conditions. Drought stress activates JA signaling, which plays a key role in plant adaptation by influencing root development. JA was found to promote xylem differentiation in roots by inhibiting cytokinin activity, suggesting a level of regulation between JA and cytokinins. Drought can also induce xylem development, potentially through the same hormonal mediation. Thus, it may be that the antagonistic interactions between JA and cytokinins are fundamental to how plants alter root architecture in response to limited water availability (Jang and Choi 2018). In their study, de Ollas et al. (2013) examined the hormonal response of citrumelo CPB 4475 citrus rootstock to extreme water deficit, focusing specifically on JA and the plant hormone abscisic acid (ABA). Hormone profiling showed that JA levels increased rapidly in a transient manner within mere hours of drought stress and then ABA levels increased relatively slowly thereafter. These studies suggest that JA acts as a regulatory hormone to trigger ABA biosynthesis and also suggest that there is some biosynthetic interaction that allows plants to effectively orchestrate multiple responses to environmental stress. Both JA and ABA are key hormones that promote plants' ability to mitigate drought or abiotic stress. In a study conducted by Hassanein et al. (2009) the effects of JA, ABA, and a combination of both hormones were applied to drought‐stressed soybean plants. Results showed that both hormones improved stress tolerance by influencing hormone levels, protein content, and polyamine accumulation.

Plants exhibit adaptive strategies in response to drought and drought‐induced oxidative stress through the phenylpropanoid pathway, which generates secondary metabolites including plant hormones and phenolics and flavonoids with powerful antioxidant action (Ahmed et al. 2021). Phenolic compounds play a key role in modulating plant responses to environmental stresses (Habibi et al. 2022). Under drought stress, increased production of phenolic compounds is generally an important component of plant protection (Faizy et al. 2025). Overall, JA induced the growth and development of Russell's plants and some phenolic compounds in response to drought, indicating a moderating role in resilience to drought stress.

## 5 Conclusion

In conclusion, results elucidate the complex and multifaceted role of JA in modulating the growth, yield components, and biochemical responses of roselle plants subjected to varying levels of drought stress. While drought stress generally exerts negative impacts on morphological and yield traits, the application of JA elicited both beneficial and detrimental effects, contingent upon the intensity of the stress and the applied JA concentration. Notably, JA frequently played a palliative role under moderate to severe drought stress by enhancing specific growth parameters (plant height, branch number), yield attributes (number of seeds per plant, calyx yield), and antioxidant metabolism (flavonoids, phenols, antioxidant percentage, and ascorbic acid under specific conditions). Furthermore, the results revealed intricate correlations between yield components and biochemical attributes, underscoring the interconnectedness of physiological responses under stress. The findings suggest that the exogenous application of JA presents a potential strategy for improving roselle performance under drought conditions; however, determining the optimal concentration and stress level for its application necessitates careful consideration. Future research is warranted to further elucidate the underlying mechanisms of JA action and to optimize its utilization in enhancing roselle resilience to water scarcity.

## Author Contributions

The authors conducted the experiments in collaboration and wrote the manuscript.

## Conflicts of Interest

The authors declare no conflicts of interest.

## Data Availability

All data generated during this study are included in this article.
